# Tranexamic Acid for reduction of intra- and postoperative TRansfusion requirements in elective Abdominal surgery (TATRA): study protocol for an investigator-initiated, multicenter, double-blind, placebo-controlled, randomized superiority trial with two parallel groups

**DOI:** 10.1186/s13063-024-08541-8

**Published:** 2024-10-19

**Authors:** Ulrich Ronellenfitsch, Anita Kestel, Johannes Klose, Artur Rebelo, Michael Bucher, Daniel Ebert, Rafael Mikolajczyk, Andreas Wienke, Thomas Kegel, Julian Hering, Christian Haiduk, Michael Richter, Jörg Steighardt, Erich Grohmann, Lutz Otto, Jörg Kleeff

**Affiliations:** 1 Department of Abdominal, Vascular and Endocrine Surgery, University Hospital Halle (Saale), Halle (Saale), Germany; 2 Department of Anesthesiology and Operative Intensive Care, University Hospital Halle (Saale), Halle (Saale), Germany; 3 Medical Faculty, Institute for Medical Informatics, Biometry and Informatics, Martin-Luther-University Halle (Saale), Halle (Saale), Germany; 4 Department of Internal Medicine IV (Hematology and Oncology), University Hospital Halle (Saale), Halle (Saale), Germany; 5 Transfusion Medicine Facility, University Hospital Halle (Saale), Halle (Saale), Germany; 6 Medical Faculty, Coordinating Center for Clinical Studies, Martin-Luther-University Halle (Saale), Halle (Saale), Germany; 7 Deutsche ILCO e.V., Association for Persons With Ostomies and Colon Cancer, Bonn, Germany; 8 Arbeitskreis der Pankreatektomierten e.V., Working Group of Pancreatectomized Persons, Magdeburg, Germany

**Keywords:** Tranexamic acid, Abdominal surgery, Perioperative bleeding, Blood transfusion, Patient blood management

## Abstract

**Background:**

Intra- and postoperative hemorrhage is a relevant problem in major abdominal surgery, leading to acute anemia and necessitating transfusion of packed red blood cells. It is estimated that in 30% of abdominal surgeries, intra- or postoperative transfusion is required. Transfusion potentially has detrimental health effects and poses a considerable socioeconomic burden. Tranexamic acid, a lysine analog inhibiting plasminogen activation and providing clot stability, has been used to reduce hemorrhage. While there is ample evidence in other surgical disciplines, it is almost completely lacking in abdominal surgery.

**Methods:**

This multicenter double-blind parallel group randomized superiority trial will compare tranexamic acid (loading dose 1000 mg over 10 min prior to skin incision, maintenance dose 125 mg/h continuously until skin closure or until 1000 mg have been administered) to placebo in patients ≥ 18 years undergoing elective esophagectomy, gastrectomy, colectomy, rectal resection, pancreatic resection, or hepatectomy. The primary efficacy endpoint is the intra- or postoperative transfusion of at least one unit of packed red blood cells. Key secondary endpoints are the number of transfused units per patient, estimated intraoperative blood loss, postoperative complications/mortality, length of hospital stay, operation/anesthesia time, D-dimer levels, and quality of life. Sample size calculation is based on the assumption that in the control group, 30% of patients require transfusion while the intervention achieves a risk reduction of 33%, reducing the probability to 20%. With a type one error of 5% and a power of 90%, using a two-sided *χ*^2^ test, this results in 412 patients per group. Accounting for non-compliance, 425 patients are to be randomized per group. The total trial duration will be 30 months with a recruitment period of 18 months.

**Discussion:**

If the proposed trial yielded positive results, the routine use of tranexamic acid in major abdominal surgery would be supported. This would avoid acute anemia with detrimental effects such as tissue hypoxia and organ injury, as well as the negative immediate and delayed effects of transfusions.

**Trial registration:**

EU CT Nr: 2023–509970-43–01, NCT06414031. Registered on 10 May 2024.

**Supplementary Information:**

The online version contains supplementary material available at 10.1186/s13063-024-08541-8.

## Introduction

### Background and rationale {6a}

Despite many preventive efforts, intra- and postoperative hemorrhage still constitutes a relevant problem in major abdominal surgery, requiring perioperative transfusion of packed red blood cells (PRBCs) in about 30% of abdominal resections [1]. In hepatic surgery, perioperative mortality is attributed to intra- and postoperative hemorrhage in about 6% of fatalities [2]. For gastrectomy and esophagectomy, postoperative hemorrhage occurs in up to 5% of patients and bears a failure to rescue of up to 30% [2, 3]. Intra- and postoperative hemorrhage and the subsequent transfusion requirement have a relevant socioeconomic burden. The direct cost for transfusing one unit of PRBCs in Germany is estimated at 147 Euros [4]. Moreover, blood loss is associated with increased postoperative morbidity and length of hospital stay, which leads to substantial indirect costs. Transfusion itself can negatively affect the health of transfused patients. Both immediate effects such as allergic reaction, hemolysis, volume and potassium overload, and long-term sequelae such as alloimmunization or immunomodulatory effects, which are supposed to increase the risk of cancer recurrence in oncological patients, have been reported. Whereas immediate effects occur in 5–10% of transfused patients, the increase of the odds for cancer recurrence has been estimated for example for colorectal cancer to be 40–60% [5]. A higher mortality of patients who received blood transfusions has been found in observational studies [6]. These considerations have led to the multidisciplinary concept of “patient blood management” which aims at limiting the need for allogeneic blood transfusion in all at-risk patients with the aim of improving their clinical outcomes [7].

Tranexamic acid (TXA), a lysine analogue inhibiting plasminogen activation and providing clot stability, has been used to reduce hemorrhage. There is ample evidence from randomized controlled trials (RCTs) and meta-analyses that TXA use is associated with reduced blood loss in orthopedic [8–11], cardiac [12], urological [13], and gynecological surgery [14]. For example, an RCT showed that the probability of being transfused PRBCs after surgery for hip fracture experienced a relative reduction of 22% [15]. While a meta-analysis comprising 104 RCTs on all surgical interventions found a 34% relative reduction in blood loss with only small differences between different operations [16], another meta-analysis of 129 RCTs demonstrated a 38% lower transfusion probability in patients who received TXA [17]. A systematic review and meta-analysis comprising 216 RCTs comparing TXA to placebo or no treatment showed a significant decrease in overall bleeding mortality with no increase in the risk of thromboembolic events as the main adverse event of concern [18]. The hitherto largest RCT including 9.535 patients undergoing non-cardiac surgery showed a 2.6% lower risk for a composite outcome of bleeding in patients receiving TXA. Subgroup results for abdominal surgery were however not presented [19].

There are few trials evaluating the specific effect of TXA administration in abdominal surgery. Four trials were conducted in patients undergoing liver transplantation and one in patients undergoing elective liver resection. While three trials showed a significantly lower blood loss and transfusion requirement for TXA [20–22], two underpowered trials failed to show a difference [23, 24]. In a retrospective series of patients undergoing resection of colorectal liver metastases, a 41% reduction in transfusion probability was found following TXA administration [25], whereas another retrospective series showed no differences in hemoglobin drop and transfusion probability [26]. One RCT on TXA use in pancreatoduodenectomy failed to show a reduction in estimated blood loss [27]. Ongoing RCTs on the use of TXA in abdominal surgery without available results comprise TXA application in cytoreductive surgery (NCT03646474), colorectal cancer surgery (NCT03606785), liver resections (NCT02261415), and bariatric sleeve gastrectomy (NL8029).

In summary, while there is high-level evidence that administration of TXA reduces bleeding and transfusion requirements in several surgical sub-disciplines, specific evidence for major abdominal surgery, i.e., esophagectomy, gastrectomy, colectomy, rectal resection, pancreatic resection, and hepatectomy, is lacking. To provide such high-level evidence, a confirmatory RCT is warranted.

### Objectives {7}

The primary objective of this trial is to assess whether intraoperative administration of TXA reduces hemorrhage and thus the need for intra- and postoperative transfusion of PRBCs for major abdominal operations.

The secondary objectives are to measure the effect of TXA administration on the number of transfused units of PRBCs, on the timing of possible transfusions, on the amount of intraoperative blood loss, on the operation and anesthesia time, on the blood markers hemoglobin and D-dimers and on quality of life of participants. Moreover, the safety of perioperative TXA administration is to be assessed.

### Trial design {8}

This trial has a multicenter, double-blind, placebo-controlled, 1:1 ratio parallel-group, randomized superiority design. It represents a low-intervention clinical trial according to Clinical Trials Regulation (EU) 536/2014.

## Methods: participants, interventions, and outcomes

### Study setting {9}

The study is conducted at eleven academic hospitals in Germany. A list of all currently enrolled centers can be obtained on the European Medicines Agency website and at ClinicalTrials.gov.

### Eligibility criteria {10}

#### Inclusion criteria

1. Age 18 years or above
2. Planned elective esophagectomy, gastrectomy, colectomy, rectal resection, pancreatic resection, or hepatectomy
3. Adequate renal function with serum creatinine < 250 µmol/L (2.82 mg/dL)
4. Written informed consent obtained before randomization
5. Negative pregnancy test for women of childbearing potential within 14 days of commencing study treatment. Females of reproductive potential must agree to practice highly effective contraceptive measures during the study. These comprise measures with a failure rate of < 1% per year when used consistently and correctly, such as intravaginal and transdermal combined (estrogen and progestogen containing) hormonal contraception, injectable and implantable progestogen-only hormonal contraception, intrauterine device, intrauterine hormone-releasing system, bilateral tubal occlusion; vasectomized partner, sexual abstinence (defined as refraining from heterosexual intercourse during the entire study period).

#### Exclusion criteria

1. Severe anemia, defined as a hemoglobin concentration < 8 g/dL (< 5 mmol/L) or anemia with hemoglobin concentration ≥ 8 to < 10 g/dL (≥ 5.0 < to < 6.2 mmol/l) and one or several of the following symptoms suggesting hypoxemia:
  Clinical signs of tachycardia, e.g., resting heart rate > 100 beats/min, palpitation, etc.
  Clinical signs of hypotension, e.g., resting systolic blood pressure < 100 mmHg, orthostatic dysregulation, etc.
  Clinical signs of dyspnea, e.g., speech dyspnea, resting respiratory rate > 20 breaths/min.
2. Thrombocytopenia with platelets < 60 × 109/L
3. Confirmed bleeding disorder with the need for specific preventive perioperative treatment (e.g., factor deficiency with the need for perioperative substitution)
4. A priori refusal of blood transfusions
5. Confirmed thrombophilia with a pertinent need for perioperative anticoagulation
6. Allergy/hypersensitivity to TXA
7. Recent (< 30 days) thromboembolic event
8. History of medically confirmed convulsions
9. In female subjects: pregnancy or lactation.

All participating study centers are institutions with a vast experience in conducting larger abdominal surgery comprising esophagectomy, gastrectomy, colectomy, rectal resection, pancreatic resection, and hepatectomy. All study sites need to fulfill GCP requirements.

### Who will take informed consent? {26a}

During visits to discuss and plan their operation, patients are informed about the rationale, design, and conduct of the trial by one of the investigators. Information material is handed out and all pending questions are discussed with the investigator. Patients are asked to provide informed consent to trial participation after due consideration.

### Additional consent provisions for collection and use of participant data and biological specimens {26b}

N/A. There are no planned ancillary studies.

### Interventions

#### Explanation for the choice of comparators {6b}

There is no established medical treatment for the prevention of bleeding and transfusion of PRBCs in major abdominal surgery, which could be used as a comparator. Given the double-blind design of the trial, the comparator thus needs to be a placebo (normal saline solution).

#### Intervention description {11a}

The intervention consists of 1000 mg TXA administered as a bolus over 10 min prior to skin incision and of continuous administration of 125 mg TXA/h from skin incision to skin closure with a maximum dose of 1000 mg. TXA is administered diluted in normal saline with a concentration of 20 mg/ml by using a perfusor via a central or peripheral line.

#### Criteria for discontinuing or modifying allocated interventions {11b}

In case of an urgent medical reason (e.g., intraoperative disseminated bleeding in which a therapeutic use of TXA is deemed indicated), unblinding is possible. In this case, the treating physicians are informed about the patient’s allocation to TXA or placebo and can act according to best individual therapeutic practice including discontinuing or modifying the intervention.

#### Strategies to improve adherence to interventions {11c}

Trial staff is instructed in detail about all interventions. Dosages and modes of administration are clearly displayed in all operation theatres in which trial participants are operated on. Perfusors are used to control the administration and to generate an alarm in case study medication is not properly reaching the participant. Empty vials which contained study medication are collected for monitoring purposes.

#### Relevant concomitant care permitted or prohibited during the trial {11d}

All perioperative and operative procedures are carried out according to clinical standards. Treatment with anticoagulants (e.g., platelet inhibitors, direct oral anticoagulants, vitamin K antagonists) is in principle allowed according to clinical indication. However, all anticoagulants should be paused or bridged with low molecular weight heparin during the perioperative period according to national and local guidelines. Pro-coagulation treatments such as coagulation factors, fresh frozen plasma, platelet transfusions, and Factor XIII substitution can be administered according to the individual judgment of the treating physician.

In case of a thromboembolic event, appropriate treatment should be initiated according to national and local guidelines. This might include medical treatment (e.g., fibrinolysis, anticoagulation) as well as interventional and surgical treatment (e.g., thrombectomy, embolectomy). In this case, an AE/SAE must be reported.

Detailed recommendations for when transfusions should be given are provided to all trial sites based on the cross-sectional guidelines for therapy with blood components and plasma derivates of the German Medical Association [28], thus minimizing heterogeneity across centers with regard to the primary endpoint.

Basic patient blood management is ensured for all participants. Measures include education initiatives and standard operating procedures, massive hemorrhage protocols, coagulation and transfusion algorithms, an online PBM e-learning course for all involved staff, screening for, diagnosis, and treatment of preoperative anemia, intravenous iron if oral iron is not tolerated, postponement of elective surgery until preoperative anemia has been classified and possibly treated, reduction of iatrogenic diagnostic-/surgery-related blood loss, avoiding unnecessary laboratory tests, lower frequency and amount of sampling, cessation and bridging strategies for anticoagulation and antiplatelet therapy, appropriate intraoperative approaches (meticulous hemostasis, minimally invasive surgery, and diathermy dissection). A detailed patient blood management manual will be provided to all trial sites.

#### Provisions for post-trial care {30}

There are no specific provisions for ancillary and post-trial care. In case of suffering harm from trial participation, a dedicated trial insurance will cover compensations.

#### Outcomes {12}

The primary endpoint is the need for transfusion of at least one unit of PRBCs. This is a valid and easily available functional parameter mirroring the immediate clinical implications of blood loss and has direct relevance on patients’ health status.

Secondary endpoints are the number of transfused units per participant, the time between skin incision and beginning of transfusion, the estimated intraoperative blood loss, operation/anesthesia times, D-dimer levels before and after the operation, hemoglobin levels before and after the operation, duration of hospital stay, quality of life, and postoperative complications and mortality.

The number of transfused units per participant, estimated intraoperative blood loss, and time between skin incision and beginning of transfusion represent measures for assumed reduction and deceleration of bleeding. The operation and anesthesia times might be shorter with reduced bleeding. D-dimer levels are a pro-coagulation state measure and hemoglobin levels reflect the amount of blood loss. Duration of hospital stay, quality of life, and postoperative complications and mortality mirror the clinical impact of hemorrhage and transfusion.

#### Participant timeline {13}

Schedule of enrolment, interventions, and assessments are shown in the table below.

**Table:** 

	**Study period**				
	**Screening**	**Enrolment and allocation**	**Post-allocation**		**Close-out**
**Timepoint**	*** − 14 to − 1 days***	**0**	***Operation* + *Intervention***	***1st post-operative day***	***Day of discharge, max. 30th post-operative day***
**Enrolment:**					
**Eligibility screening**	X				
**Informed consent**	X				
***Routine laboratory parameter screening***	X	(X)			
**Allocation**		X			
**Interventions:**					
***Preoperative bolus 1000 mg TXA/10 min i.v. followed by intraoperative continuous infusion of 125 mg TXA/hr i.v. or placebo (normal saline solution)***			X		
**Assessments:**					
***Routine laboratory examination***^b^	X	(X)^a^		X	X
***Safety assessments (AE/SAE/AESI)***			X	X	X
***Transfusion Necessity***			X	X	X
***Concomitant medication***			X		
***Estimated intraoperative blood loss***			X		
***Operation* + *anesthesia duration***			X		
***Number of transfused PRBCs***			X	X	X
***Time to transfusion***			X	X	X
***Postoperative complications/mortality***			X	X	X
***Duration of stay***					X
***Quality of life (SF-12)***		X			X

^a^Pregnancy test: urine or blood beta-HCG within 72 h preoperatively in women of childbearing age

^b^Routine laboratory examinations: complete blood count, sodium, potassium, calcium, creatinine, and estimated GFR (MDRD formula), bilirubin, ALAT, ASAT, INR, apTT, D-Dimer levels

#### Sample size {14}

The sample size calculation is based on the assumption derived from the literature and own hospital data that in the control group (placebo), 30% of participants require intra- or postoperative transfusion of at least one unit of PRBCs [1, 29], and that the intervention (TXA) achieves a relative risk reduction of 33% [18], reducing the probability to 20%. This difference would not only be expected, but also represents a clinically meaningful value. With a type one error (alpha) of 5% and a power of 90%, using a two-sided *χ*^2^ test, this results in 412 participants per group (Software PASS).

Given that the primary endpoint is ascertained at the end of each participant’s hospital stay, virtually no loss to follow-up is expected. Non-compliance, i.e., withdrawal of consent during the study period, i.e., the hospital stay, is expected in 3% of patients, resulting in 425 participants per group to be randomized.

#### Recruitment {15}

Previous trials conducted in fields other than abdominal surgery support the notion that the randomization of a large number of patients between receiving TXA or not is feasible in a short time frame as long as the case volume in the respective study center is sufficiently high [19]. All participating centers are high-volume institutions in abdominal surgery, having relevant case volumes. All eligible participants planned for major abdominal surgery in the trial sites will be screened for and actively offered trial participation. Thus, complete trial recruitment within 18 months is deemed feasible.

### Assignment of interventions: allocation

#### Sequence generation {16a}

Participants will be randomized within 24 h prior to surgery in a 1:1 ratio to the TXA or the placebo group. Randomization will be stratified by planned surgical access (open vs. minimally invasive) and planned operation (esophagectomy, gastrectomy, colectomy, rectal resection, pancreatic resection, or hepatectomy). Randomization will be done via an online tool (secuTrial®) generating a randomization number.

#### Concealment mechanism {16b}

Randomization via an online tool (secuTrial®) generating a randomization number will ensure that group assignment is unbiased and concealed from participants, treating physicians, investigators, and other study staff. The online tool will provide the randomization number, which is identical to the Study Medication Number/reference code of the investigative medicinal product (IMP) label. All vials with study medication look identical, so that it remains concealed if participants receive TXA or placebo.

#### Implementation {16c}

The online randomization tool (secuTrial®) will generate the allocation sequence and assign participants to interventions by means of providing a randomization number. Participants will be screened and enrolled by investigators at the trial sites. The assignment of participants to the intervention or control arm will be blinded and done by randomization via the online randomization tool.

### Assignment of interventions: blinding

#### Who will be blinded {17a}

Trial participants, treating physicians, investigators, and other study staff will all be blinded regarding assignment to interventions.

#### Procedure for unblinding if needed {17b}

Only in case of an urgent medical reason (e.g., intraoperative disseminated bleeding in which a therapeutic use of TXA is deemed indicated), unblinding is possible. Such unblinding can be done by opening the appropriate blinding envelope upon due justification by the site investigator. The sponsor must be informed by fax about unblinding and its justification immediately (within 24 h). After unblinding, the treating physicians are informed about the participant’s allocation to TXA or placebo and can act according to the best individual therapeutic practice.

### Data collection and management

#### Plans for assessment and collection of outcomes {18a}

All data of trial participants are assessed and collected by investigators and study nurses. Study visits take place upon recruitment (14 days to 1 day prior to surgery), on the day of surgery, on day 1 after surgery, and on the day of hospital discharge or postoperative day 30, whatever occurs first. Trial staff is specifically trained for data assessment and collection. Data on the primary endpoint, PRBC transfusion, is extracted from specific transfusion files and patient files or hospital information systems. Laboratory data is extracted from the laboratory systems of the trial sites. Validation certificates for the single laboratories need to be provided. Clinical data including intraoperative data are collected from patient files or hospital information systems. Quality of life is measured with the validated German version of the SF-12 questionnaire. The questionnaire is handed out to trial participants and collected by study staff after it has been filled by the participant. All collected data will be documented in the study-specific electronic case record form (eCRF) as promptly as possible. Any entry in the eCRF has to be traceable to the information contained in the source data. eCRF templates are available upon request from the principal investigator.

#### Plans to promote participant retention and complete follow-up {18b}

Given that the primary endpoint is ascertained at the end of each participant’s hospital stay, virtually no loss to follow-up is expected. Non-retention, i.e., withdrawal of consent during the hospital stay is not expected in a relevant proportion of participants. If participants decide to withdraw from the trial, complete collection and documentation of outcome data will still be sought if the participant agrees.

#### Data management {19}

Investigators or designated persons will document the study data in the study-specific eCRF as promptly as possible. Any entry in the eCRF has to be traceable to the information contained in the medical file and the source data. The data will be entered directly at the study site by the site staff via remote data entry in the study-specific eCRF in English. The study management software secuTrial (iAS GmbH, Berlin, Germany), a validated database-driven complete solution for the conduction of clinical studies, will be used for data entry and query management (cooperation between Coordination Center for Clinical Trials [KKS] Halle and Center for Clinical Trials [ZKS] Leipzig).

Management of the study data base will be done by KKS Halle. All changes in the data will be recorded by an audit trail. The study software provides an adaptive concept for user accounts and user roles depending on the study. The data base is integrated in a general IT infrastructure and security concept including a firewall and backup system. The data will be saved on a daily basis by ZKS Leipzig. In a multistage procedure, the obtained data will be checked electronically for their plausibility and consistency by KKS Halle. In case of implausible or missing data, queries will be generated and provided to the investigator in order to obtain any missing information and resolve inconsistencies. After completion of data capture and quality check the data base will be closed by KKS Halle and the data will be transferred to the biometrician for statistical analysis.

#### Confidentiality {27}

Information about study patients will be kept confidential and managed under the applicable laws and regulations. All investigators must ensure data protection of the patients; Patients must not be identified by names in any documents submitted to the sponsor. Signed informed consent forms and patient identification logs must be kept strictly confidential to enable patient identification at the site. All study-related information is stored securely at the study site. On any document that is submitted to the sponsor, subjects shall be identified by the unique Patient-ID only (pseudonymized). It must be ensured that the Sponsor and KKS Halle do not receive any data that would allow conclusions to be drawn about the identity of the person concerned.

#### Plans for collection, laboratory evaluation, and storage of biological specimens for genetic or molecular analysis in this trial/future use {33}

Not applicable. The trial design does not foresee any collection of biological specimens.

### Statistical methods

#### Statistical methods for primary and secondary outcomes {20a}

The primary endpoint will be compared in the intention-to-treat population between the two study arms with a logistic regression model including the treatment group, and the stratification variables type of operation (esophagectomy, gastrectomy, colectomy, rectal resection, pancreatic resection, hepatectomy) and type of planned surgical access (open, minimally-invasive) as covariates.

The secondary endpoints of postoperative in-hospital complications and mortality will be analyzed similarly. The number of transfused units of PRBCs, estimated intraoperative blood loss, length of hospital stay, operation time, anesthesia time, D-dimer levels, and quality of life will be analyzed using a linear regression model including treatment group, type of operation, and type of surgical access as covariates. In case of heavy skewness in the outcome data, the outcome variables will be transformed to reduce skewness. All results for secondary outcomes will be interpreted in an exploratory sense. Dichotomous outcomes will be reported as absolute and relative frequencies and continuous variables by means, medians, standard deviations, and ranges. Time to transfusion will be compared between both arms using time-to-event analysis.

A safety analysis will be carried out by comparing the frequency of all adverse events and adverse events attributed to the intervention between the two study arms. Patients with a history of thromboembolic events will be analyzed as a subgroup of special interest.

#### Interim analyses {21b}

If deemed necessary after due consideration by all of its members, the data safety monitoring board (DSMB) may initiate an unplanned interim analysis regarding safety and efficacy at any time point during the trial. The DSMB will then give recommendations to the principal investigator for continuation, discontinuation, modification, or termination of the study.

The Sponsor, the authorities, and the competent ethics committee have the right to discontinue the trial prematurely in consequence of the results of the interim analyses if a new finding concerning significant superiority or inferiority in one of the treatment arms, with regard to the primary endpoint data, arises or if unacceptable risks and toxicities have occurred.

#### Methods for additional analyses (e.g., subgroup analyses) {20b}

Subgroup analyses will be conducted according to surgical access (open vs. minimally-invasive), kind of operation (esophagectomy, gastrectomy, colectomy, rectal resection, pancreatic resection, or hepatectomy), and for patients with a history of a thromboembolic event.

#### Methods in analysis to handle protocol non-adherence and any statistical methods to handle missing data {20c}

The primary analysis data set is the intention-to-treat population. This data set contains all patients who have been enrolled in the clinical trial and randomized. The secondary analysis data set is derived from the per-protocol population. This data set includes all patients who have been treated according to the protocol during the whole duration of the study and reached a defined endpoint. The tertiary analysis data set (safety population) contains all patients who have received at least one dose of study medication. There are no specific statistical methods stipulated to handle missing data, because the proportion of patients with missing endpoint data is expected to be very low.

#### Plans to give access to the full protocol, participant-level data, and statistical code {31c}

Access to the full study protocol and statistical code will be granted upon reasonable request to the principal investigator. In accordance with data protection regulations, de-identified individual participant data will be shared with other researchers upon reasonable and justified request. It is planned to store de-identified individual participant data on the B2SHARE repository of the EUDAT collaborative data infrastructure.

### Oversight and monitoring

#### Composition of the coordinating center and trial steering committee {5d}

All trial activities are managed by a trial steering committee consisting of the principal investigator, two more authors of the study protocol, and two representatives from participating study sites. The trial steering committee decides, among other issues, about the recruitment and initiation of additional study centers and the modification to the study protocol following DSMB advice or due to other requirements. The committee convenes every 3 months during the entire trial period.

KKS Halle supports the trialists in all matters concerning the clinical trial including administrative project management, data management, monitoring, safety management, statistical analysis, and the performance of administrative tasks and duties.

#### Composition of the data monitoring committee, its role and reporting structure {21a}

An independent Data Safety Monitoring Board (DSMB) has been established to monitor safety data during the course of the clinical study. It comprises one expert each from surgery, anesthesiology, transfusion medicine, and biostatistics as well as a patient representative. The DSMB will receive regular information on safety events occurring in trial participants, in particular details on all reported SAEs, every 3 months during the trial period, and then convene for virtual meetings. This anonymized information will be prepared by the KKS. Moreover, if deemed necessary after due consideration by all of its members, the DSMB may initiate an unplanned interim analysis regarding safety and efficacy at any time point during the trial. The DSMB will give recommendations to the coordinating investigator for continuation, discontinuation, modification, or termination of the study.

All members of the DSMB are completely independent of the trial steering committee and investigators participating in the conduct of the trial. Their work is not subject to any directives by the aforementioned persons. In addition to its safety tasks, the DSMB provides the funding agency every 6 months with information about the trial progress and, if deemed necessary, provides advice regarding modifications of the original funding and time plan.

The composition and responsibilities of the DSMB and the structure and procedures of its meetings are laid down in a DSMB charter, which is available upon request from the principal investigator.

#### Adverse event reporting and harms {22}

Any adverse event (AE) occurring from IMP administration to the end of follow-up has to be documented. All AEs have to be followed until resolution/stabilization, improvement is not to be expected or end of study for the respective patient. AEs have to be recorded in the patient records and eCRF. The investigator is responsible for the assessment of the AE regarding severity, seriousness, and causal relationship to the IMP. Therefore, the following has to be documented:

- Event (diagnosis/symptom/results, etc.) with start date (onset) and end date (or ongoing on final examination at the end of the study), date of awareness
- Severity according to the current version V5.0 of CTCAE:

Grade 1—mild: asymptomatic or mild symptoms, clinical or diagnostic observations only; intervention not indicated.

Grade 2—moderate: minimal, local, or non-invasive intervention indicated; limiting age-appropriate instrumental activities of daily living (ADL).

Grade 3—severe: medically significant but not immediately life-threatening; hospitalization or prolongation of hospitalization indicated; disabling; limiting self-care ADL.

Grade 4—life-threatening: urgent intervention indicated.

Grade 5—death: death related to AE.

- Causality—relationship to the IMP (only TXA arm) according to the investigator (related/not related): Reasonably possible/related: The event occurs with a reasonable time relationship to study medication intake. There is no reasonable explanation that the event is caused by disease or other drugs. Reasonably NOT possible/not related: The event occurs with a time relationship to study medication intake that makes a relationship improbable. Diseases or other drugs provide plausible explanation
- Serious/non-serious. If the event meets any of the seriousness criteria additional documentation and reporting is required (see below)
- Actions taken for AE itself and regarding the IMP (including temporary or permanent)
- Outcome of the event

For the classification of the adverse events, the MedDRA code system will be used by KKS Halle.

A serious adverse event (SAE) has to be documented if it occurred between IMP administration and the end of follow-up. Serious adverse reactions (suspicion for causality related to the IMPs) have to be documented and reported over the entire course of the study including the follow-up period (until Visit 3). Additionally, an SAE has to be reported immediately (i.e., within 24 h upon becoming aware of the event) to KKS Halle using a SAE reporting form. Additional information including the course and outcome of the event can be completed in the follow-up report (an identical form can be used). The investigator will provide additional information if requested by the ethics committee, the competent federal authority, or the sponsor. Details are provided in an SAE reporting manual.

#### Frequency and plans for auditing trial conduct {23}

All investigators will permit study-related monitoring and audits, ethics committee review, and regulatory inspections, providing direct access to source data/documents as ensured by the investigator’s agreement and the cooperation agreement between the sponsor and KKS Halle.

Monitoring will be done in cooperation with the KKS Halle according to ICH-GCP E6 and standard operating procedures (SOPs). Patient recruitment will begin after the initiation visit and once all essential documents are available. Monitoring will be done centrally (check of the data entered into the eCRFs by KKS Halle) and by means of on-site visits at the respective study site. The frequency of on-site visits depends on the patient recruitment. The monitor’s access to the study documents and medical records is ensured by the investigator’s agreement and the cooperation agreement between the sponsor and KKS Halle. The KKS Halle guarantees that patient confidentiality will be respected at all times. Participation in this study will be taken as an agreement to permit direct source data verification. Initially, a formal check of captured information for completeness and plausibility will be done. Thereafter, a check of the correct transfer of data from the source data will be done. The informed consent forms, the inclusion and exclusion criteria, and the data related to the primary target variables will be checked completely (100% source data verification). The specific extent of the monitoring and the source data verification are specified in a monitoring manual.

To ensure quality of data and study performance the sponsor may conduct site visits by an independent auditor. An audit will only be performed after notification and arrangement with the investigator. An audit certificate will be issued as quality proof. There is a possibility of inspections by the responsible supervisory authority for the purpose of supervision of the ongoing or completed clinical study. If an inspection of the study site is announced by the authority, the investigator must inform the sponsor immediately. The investigator ensures immediate access to any study-related documents including the original data if requested by a representative of the sponsor (monitor and auditor), the ethics committee, or the responsible authorities.

#### Plans for communicating important protocol amendments to relevant parties (e.g., trial participants, ethics committees) {25}

The sponsor may make modifications to the protocol. Substantial modifications according to Regulation (EU) 536/2014 have to be submitted through the EU portal by the sponsor and approved by the competent authority and ethics committee before being implemented. Only changes to the protocol that are required for patient safety may be implemented prior to the competent authority and ethics committee approval. Modifications will be signed by all signatories of the protocol. All investigators will acknowledge the receipt and confirm by their signature that they will adhere to the amendment. The site investigators will be responsible for implementing any modifications at the study site (including the distribution of modifications to all staff concerned).

#### Dissemination plans {31a}

Results of the trial shall be disseminated directly to decision-makers such as surgeons, anesthesiologists, transfusion medicine specialists, etc. by means of publication in a peer-reviewed journal. If possible, protocol and publication will be published open access. The means of dissemination will be presentations at national and international scientific conferences as well as specific events. In particular, a virtual or on-site symposium at which the results of the analysis will be presented and discussed among decision-makers is planned. Results will be actively presented to the bodies in charge of national and international treatment guidelines (e.g., Association of the Scientific Medical Societies in Germany [AWMF], German Society of General and Visceral Surgery [DGAV], and German Society of Anaesthesiology and Intensive Care Medicine). Because results are expected to have a direct and relevant impact on patients’ decision-making, they will be specifically communicated to patients and the general public. Possible media of dissemination are health-specific sections of newspapers, radio, and TV programs as well as a direct approach through the patient organizations involved in the trial.

The patient organizations Deutsche ILCO e.V. (representing patients with ostomies and/or colorectal cancer) and Arbeitskreis der Pankreatektomierten e.V. (representing patients who underwent pancreatic surgery) have been closely involved in the conceptualization of the trial. In particular, the treatment modalities, trial design, and the primary endpoint have been discussed with representatives of these organizations. Both organizations endorse the trial concept and are continuously involved during trial conduction. The patient organization Lebertransplantierte Deutschlands e.V. (representing patients after liver transplantation) is represented in the DSMB of the trial.

Patient involvement is crucial in order to define the relevance of endpoints to patients. The defined endpoints were the basis of discussion with patient representatives prior to the application for approval by authorities. A focus group discussion with at least five patient representatives (one each representing patients undergoing esophagectomy, gastrectomy, colorectal resections, hepatectomy, and pancreatic resections) will be conducted during recruitment. This discussion serves to rank the subjective importance of the assessed endpoint to patients prior to publication. During the recruitment phase, patient organizations will be available for pending questions of the trial team or patients. After completion of the trial and data analyses, results will be discussed again in the framework of a focus group discussion with five patient representatives. Similar to the first endpoint discussion, the importance of the results of the single endpoints will be ranked. Both rankings will be reported in all presentations of results. Patient organizations will also be appropriately represented in the writing and publication of trial reports and scientific publications.

## Discussion

Intra- and postoperative hemorrhage is a relevant problem in major abdominal surgery, leading to acute anemia and necessitating transfusion of PRBCs. It is estimated that in 30% of abdominal surgeries intra- or postoperative transfusion is required. Transfusion potentially has detrimental health effects and poses a considerable socioeconomic burden. TXA, a lysine analogue inhibiting plasminogen activation and providing clot stability, has been used to reduce hemorrhage. While there is ample evidence in other surgical disciplines, it is almost completely lacking in abdominal surgery. This multicenter double-blind parallel group randomized superiority trial compares TXA to placebo in patients ≥ 18 years undergoing elective esophagectomy, gastrectomy, colectomy, rectal resection, pancreatic resection, or hepatectomy. The primary efficacy endpoint is the intra- or postoperative transfusion of at least one unit of PRBCs.

If the proposed trial yielded positive results, the routine use of TXA in major abdominal surgery would be supported. Minimizing hemorrhage in patients undergoing major abdominal surgery would avoid acute anemia with its detrimental effects such as tissue hypoxia and organ injury, as well as the negative immediate and delayed effects of necessary transfusions of PRBCs such as allergic reactions, immunization, and an increased risk of tumor recurrence in certain cancers. Time to recovery from surgery and length of hospital stay would be shortened. If the efficacy of the administration of TXA for the reduction of hemorrhage and transfusion requirements could be demonstrated, the drug would most likely become a new standard treatment during major abdominal surgery. While the drug itself is cheap, a favorable socioeconomic impact could be achieved by saving costs for transfusions and avoiding bleeding- and transfusion-related complications leading to prolonged hospital stay and absence from work or disability.

## Trial status

Protocol version 2.2, July 4, 2024. Recruitment began: June 17, 2024. Expected recruitment completion: November 2025.

## Supplementary Information

Supplementary Material 1

## Data Availability

All trial investigators will have access to the final trial dataset. In accordance with data protection regulations, individual de-identified individual participant data will be shared with other researchers upon reasonable and justified request. It is planned to store de-identified individual participant data on the B2SHARE repository of the EUDAT collaborative data infrastructure.

## Abbreviations

AE: Adverse event
DSMB: Data safety and monitoring board
eCRF: Electronic case record form
IMP: Investigative medicinal product
KKS Halle: Coordination Center for Clinical Trials Halle
PRBCs: Packed red blood cells
RCT: Randomized controlled trial
SAE: Serious adverse event
TXA: Tranexamic acid
ZKS Leipzig: Center for Clinical Trials Leipzig
