# Supplementary material for: Tranexamic Acid for reduction of intra- and postoperative TRansfusion requirements in elective Abdominal surgery (TATRA): study protocol for an investigator-initiated, multicenter, double-blind, placebo-controlled, randomized superiority trial with two parallel groups
Source: Trials. 2024 Oct 19;25:695. doi: 10.1186/s13063-024-08541-8 (PMC11490177; doi:10.1186/s13063-024-08541-8)
Supplement: Supplementary file 1 — Supplementary Material 1 [file 13063_2024_8541_MOESM1_ESM.pdf]

DLR Project Management Agency

Heinrich-Konen-Straße 1

53227 Bonn

Martin-Luther-Universität Halle-Wittenberg

Magdeburger Str. 8

06112 Halle (Saale)

Grant notification

SUBJECT Grant from the federal budget, Section 30, Chapter 04, Title 68530,

Financial year 2023, for the project:

"TATRA - Tranexamic Acid for reduction of intra- and postoperative transfusion requirements in elective abdominal surgery: randomized controlled trial"

Executive institution: Martin-Luther-University Halle-Wittenberg - Medical Faculty and

University Hospital - University Clinic and Polyclinic for Visceral, Vascular and Endocrine Surgery

Funding reference: **01KG2305**

REFERENCE

Your application dated 27.11.2022, with supplement of 10.01.2023 (e-mail), 13.01.2023 (e-mail),16.01.2023, 01.02.2023 (e-mail), 13.03.2023 (e-mail), 15.03.2023 (e-mail), 21.03.2023, 29.03.2023 (e-mail)

ANNEX

- Reproduction of "Ancillary provisions for grants on an expenditure basis of the Federal Ministry of Education and Research for project funding (NABF)" (status: December 2022)

- Copy of the "Special ancillary provisions for the call-off of grants in the call procedure in the business area of the BMBF - BNBest-mittelbarer Abruf BMBF" (Status: January 2015)

- Overall financing plan

- Confirmation of receipt" form

- "Waiver of legal remedies" form

- Form "Application profi Online"

- "Payment request" form with instructions for payment recipients

- Sample list of receipts as an attachment to the proof of use

- Sample interim report for projects according to NABF

- Sample final report for projects according to NABF

- Form "Declaration (ICH-GCP)"

- Form "Declaration DSMC"

- Form "Sample for graphical representation of patient recruitment"

Dear Sir or Madam,

1. amount of the grant/form and type of funding/type of earmarking/period of approval/ payment schedule

On behalf of and with funds from the Federal Ministry of Education and Research (BMBF), we grant you, as the authorised project executive agency, a non-repayable grant of up to grant of up to € 1,490,254.09 (grant without project lump sum) up to a maximum of amount of the eligible expenditure (full funding) plus a project lump sum of € 298,050.82 (20.00 % of the above-mentioned grant or of the eligible expenditure financed by the BMBF).

The total grant including the project lump sum therefore amounts to

1.788.304,91 €

(in letters: one-seven-eight-eight-three-zero-four-point-nine-one euro).

The above amount is a maximum amount ("up to"/"maximum"), i.e. the specific amount of the grant will only be determined after the proof of use to be submitted by you and is subject to reservation until then.

The decision which expenses are to be recognized as eligible for funding within the scope of the is based on the regulations set out in this grant notification and the associated ancillary provisions. In the final amount of the grant, additional cover funds within the meaning of No. 2 NABF are given priority over the grant on the basis of the principle of subsidiarity and therefore have the effect of reducing the grant, if necessary on a pro rata basis.

The grant is earmarked; it may only be used for the above-mentioned project in accordance with your application dated 27.11.2022, including any supplements (see reference) and the attached application form amended by the attached overall financing plan amended by us in agreement with you.

Approval is conditional on the overall financing of the project remaining secured.

The grant is valid for the period from 01.04.2023 to 30.09.2025 (approval period).

The grant may only be settled for the expenditure incurred for the project during the approval period.

We intend to make the grant, including the project lump sum, available in cash as follows:

€ 235,351.01 in the financial year 2023

€ 965,268.59 in the financial year 2024

€ 587,685.31 in the financial year 2025.

With regard to the efficient management of the total federal funds available to us for this area, the originally planned budget was adjusted as follows.

From the first year of the term, 25% of the annual amount and, in the following year, 10% of the of the annual amount will initially be transferred to the end of the term. The amount of the total grant, the overall financing plan and the approval period remain unaffected by this measure. We assume that the work plan and schedule can be adhered to as approved.

If the federal funds available to you in the respective financial years are not sufficient, we will, within the scope of the possibilities available to us, attempt to adjust the use of funds to meet your needs by bringing forward the funds, based on your request, which must be submitted in good time. If the need for funds is delayed compared to your application, this must be requested immediately (by October 15th of each financial year at the latest), attaching new financing plans for the relevant financial years, so that an attempt can be made to adjust the payment plan.

2. Additional provisions and information

The attached NABF, which binds and authorizes you as the grant recipient directly towards us, are part of this notice in accordance with the following provisions. The BMBF reserves the right to exercise the rights arising from them independently or jointly with us.

The call procedure according to No. 2.5.1 NABF applies to the payment of the grant. The enclosed BNBest-mittelbarer Abruf BMBF are part of this notice in accordance with the following provisions.

The project lump sum will be paid by means of a payment request amounting to 20% of the eligible expenditure financed by the BMBF. The numerical proof must confirm the amount of the project lump sum for the indirect project expenditure. The actual amount of the grant, which results after checking the proof of use, is decisive for the final determination of the amount of the project lump sum.

The following additional ancillary provisions and information apply:

Avoiding cross-subsidization

To avoid cross-subsidization, you are obliged to prove the clear separation of economic and non-economic activities and their expenditure at the latest with the proof of use, e.g. in the annual financial statements. (see No. 2.1.1 of the EU Commission's Union Framework for State Aid for Research, Development and Innovation of October 28, 2022).

Note on state aid law

The classification under state aid law is based in particular on your related information in the funding application. It must be ensured that this information is complete, correct and up-to-date. You are obliged to immediately report any changes to this information that are relevant to state aid law. This obligation exists regardless of the - punishable - obligation to report facts relevant to the subsidy.

Subsidized nature of the grant

The grant is a subsidy within the meaning of Section 264 Paragraph 8 No. 1 of the Criminal Code (StGB). You were informed in a letter dated December 14, 2022 of the facts relevant to the subsidy and the criminal liability of subsidy fraud under Section 264 of the Criminal Code and confirmed this in a letter dated January 16, 2023. The content of this correspondence will become part of the grant decision.

Facts that conflict with the approval, granting, continued granting, use or retention of the subsidy or subsidy benefit or that are significant for the reclaiming of the subsidy or subsidy benefit must be reported immediately. Special disclosure obligations remain unaffected.

Payment blocks

The grant for the following individual items of the overall financing plan will be blocked: Funds for the external study centers (item F0843 - case fees of the overall financing plan) amounting to €726,660 are initially blocked. They can be unblocked as follows depending on the progress of the study:

These funds can be unblocked as follows depending on the progress of the study:

€110,100 if the first external patient is included

€183,500 if 183 external patients are included

€183,500 if 367 external patients are included

€146,800 if 550 external patients are included

€102,760 if 734 external patients are included

The inclusion of the corresponding number of external patients must be confirmed to us by the person responsible for the database.

Blocked federal funds cannot be paid. You can only access blocked funds after a written notice of change. We reserve the right to reduce the blocked federal funds. Items affected by the block are excluded from exchangeability in favor of other items in the overall financing plan.

Changes to the overall financing plan

Changes to the overall financing plan that go beyond the authorization of No. 2.1.1 NABF require our prior written consent. A new version of the overall financing plan must be attached to the application.

Right of revocation

We reserve the right to revoke the decision

- in the cases of No. 6.1 NABF,

- in the case of a payment block for individual items in the overall financing plan,

- for compelling reasons

and to stop the funding in whole or in part (right of revocation according to Section 36 Paragraph 2 No. 3 in conjunction with Section 49 Paragraph 2 No. 1 of the Administrative Procedure Act).

Personnel expenses

1. The salary groups/wage groups on which the personnel estimates in the attached overall financing plan are based represent the upper limit of eligibility for funding (except for a promotion based on probation in line with the collective agreement). However, this does not release you from the responsibility for collective agreement-compliant classifications and remuneration/wages. Grants, holiday pay and personnel-related material expenses (e.g. separation allowances, relocation expenses) are only eligible for funding if they are paid within the approval period. If employees are entitled to an annual special payment, this can only be recognized as eligible for funding in accordance with the proportion of the costs incurred in the

approval period of the project in question (x/12; so-called twelfth rule) and provided that the payment was made no later than 6 months after the end of the approval period.

2. In order to fulfill the obligation pursuant to No. 4.7 NABF, the actual proportionate use of the employees estimated in the financing plan must be regularly updated on a project-related basis so that the personnel expenses can be recorded in the numerical evidence in accordance with the costs incurred.

3. For people who work part-time for the elderly in the block model, the eligible personnel expenses are to be determined as follows: For the active phase, fictitious salary components are not eligible for funding. In the passive phase, the personnel expenses incurred can be settled up to the end of the approval period. Funding in the passive phase is limited to the difference between full remuneration and the expenses that were eligible in the active phase. After the project funding has ended, further funding is not possible. In the case of partial retirement in the part-time model, only expenses for work that benefits the project are eligible.

4. The determined personnel expenses for people not exclusively employed in the project may only be used pro rata.

5. If you as an employer are obliged to pay the U 1 levy (compensation procedure for continued payment of wages in the event of illness), in principle only the statutory minimum rate (reduced levy rate) is recognized as eligible. Health insurance benefits as continued payment of wages are to be credited pro rata to the personnel expenses recognized as eligible.

Subcontracting

In addition to the regulations in the NABF, the following applies:

Contracts up to a maximum value of €30,000 (excluding VAT) may be awarded by way of negotiated award in accordance with Section 8 Paragraph 4 No. 17 UVgO in compliance with the following provisions, without any further reasons under Section 8 Paragraph 4 UVgO being required.

In order to ensure the economic efficiency of the award, the following implementation provisions must be observed:

- Deliveries and services up to an estimated contract value of €1,000 (excluding VAT) can be procured without a tendering procedure, taking into account the principles of economic efficiency and economy (direct contract in accordance with Section 14 UVgO)

- Deliveries and services with an estimated contract value of €1,000 (excluding VAT) to €30,000 (excluding VAT) can be awarded by way of negotiated award after obtaining at least three written offers.

- For deliveries and services with an estimated order value of €10,000 (excluding VAT) to €30,000 (excluding VAT), a written request to submit an offer (including a description of the service) is also required. The awarding of contracts must be documented in accordance with Section 6 UVgO. The admissibility of awarding contracts by way of negotiated award in accordance with Section 8 Paragraph 4 Numbers 1 to 16 remains unaffected. Companies that are invited to submit an offer or to participate in negotiations should be rotated. No. 2.4 NABF must also be observed if potential contractors have already been named or offers submitted in the funding application.

Travel

For necessary trips to non-European countries that were not listed/justified in detail in the application (e.g. location, period, [conference] program), our prior consent must be obtained. Without prior consent, these trips cannot be considered as eligible for funding.

Information for payment recipients

The "Information for payment recipients" attached to this notice must be observed.

Participation in "profi-Online"

You have the option of participating in the semi-electronic hybrid procedure "profi-Online". Relevant information and an application are attached to this notice. If you are interested in participating in "profi-Online", please send the completed application to the project agency. This agency is also available for more detailed information about the procedure. For projects that are approved on the basis of the BNBest indirect call-up BMBF, the “profi-Online” procedure is mandatory.

Proof of use

The proof of use consists of a factual report and the numerical proof in accordance with No. 4.1 NABF. Factual reports must also take into account the requirements of No. 4.2 NABF. The numerical proof of use must be accompanied by a tabular overview of documents (list of documents according to the enclosed template) in accordance with No. 4.3 NABF. You will receive a DV form for the numerical proof after the end of the approval period. When submitting an interim or proof of use in accordance with 4.4 or 4.1 NABF, the respective template for an interim or final report attached to this grant notification as an appendix must be used. The numerical interim and usage proof must be calculated by an authorized person.

According to No. 4.9 NABF, the numerical usage proof must be checked by your auditing institution. The result of the check must be noted and certified on the usage proof.

Publications

1. In addition to No. 5.2.2 NABF, the BMBF logo with the addition “Funded by” must be clearly visible on publications and public relations measures – for example trade fairs, websites or others. The logo as well as further information on observing the logos and corporate design of the BMBF funding body can be accessed at the URL http://www.bmbf.de/bmbfservice/4607.php with the username: “zuwendungs-info” and the password “XXXXXXXX”.

2. If you publish the results of the research project as an article in a scientific journal, free electronic access (open access) to the article should be possible. If the article does not initially appear in a journal that is electronically accessible free of charge, you should also make the article electronically accessible free of charge - if necessary after expiry of a reasonable period (embargo period - secondary publication). In the case of secondary publication, the embargo period should not exceed twelve months.

3. When publishing on the Internet with the establishment of an Internet address, the following must be observed:

3.1 Registration

The start URL and, if applicable, the Internet domain of the websites created for the project must be reported to the responsible department / project sponsor. The registration should contain the funding code in addition to the URL.

3.2 Deregistration, domain abandonment

If continued use of an internet domain for project purposes in the sense of exploiting the results is not pursued or is no longer pursued at a later date and you want to abandon the internet domain secured for a project after the end of the project or at a later date, the BMBF must be informed of this by email at website@bmbf.bund.de before returning the domain so that the BMBF can decide whether to take over the domain to be abandoned in individual cases. If the BMBF takes over a domain in individual cases, you must hand it over to the BMBF free of charge and cooperate in the transfer (KK application).

Public relations

Science and research depend on broad acceptance at all levels of society. In order to achieve a positive basic attitude among the population towards the concerns and needs of research, it is important to maintain contacts between science and the public and to intensify communication. All scientists are called upon to make their contribution to this. They are therefore obliged to inform the public about the goals and results of this project on suitable occasions. Examples of possible options include publications in popular science journals, reports or interviews in the daily press or radio media, active participation in public information events, open days, Internet presentations or similar; the BMBF must be named as the sponsor in each case. Such public relations activities should take place at least once a year. This must be reported on in the regular interim reports and the final report.

Data protection

To ensure data protection, the necessary data protection measures must be taken in due course and, in cases of doubt, the bodies responsible for data protection control (in the public sector, the state data protection officers and the federal data protection officer, otherwise the company data protection officers) must be involved.

Documents that must be submitted before the first patient is included

- The unconditional positive vote of the responsible ethics committee for the study " TATRA - Tranexamic Acid for reduction of intra- and postoperative transfusion requirements in elective abdominal surgery: randomized controlled trial " and the approved version of the study protocol in electronic form (e.g. as a PDF)

- Confirmation of registration of the study in a WHO-certified primary registry (e.g. German Register of Clinical Studies; DRKS) with the registration number. The deposited data set must contain the funding code and, if possible, also name the BMBF as the funding organization. The data set must be continuously updated during the course of the project.

- The legally binding signed commitment to the guideline for good clinical practice (ICH-GCP); the form for the declaration is enclosed. The declaration must be submitted to us in original.

- A declaration from all members of the independent data safety monitoring board (DSMB). The declaration must be made on the enclosed form and submitted to us.

We ask that you inform us by email about the status of the above-mentioned documents by August 31, 2023 at the latest. These documents are mandatory for the funding of the project.

The grant notification contains a general right of revocation. If the documents are not complete by the above-mentioned date, we will immediately examine the revocation of the grant.

Before the first patient is included, our consent to start recruitment is required.

Involvement of patients

The needs of patients must be taken into account appropriately, and patients or their representatives must be involved in all projects in an appropriate manner. We ask that you provide us with the names of the patients, patient representatives or patient organizations involved in the study by August 31, 2023 at the latest. In addition, the implementation of the planned participation of patients must be presented in the annual interim reports and in the final report.

Publication of the study protocol and the study results

In order to achieve transparency about the research carried out, you are obliged to publish the study protocol including all documentation forms (CRF) in a relevant scientific journal by August 31, 2023. In addition, the results of the study must be entered into a WHO-certified primary register within one year of the database being closed. In addition, the results of the study must be published within another year. This includes at least the publication of the results at a scientific congress and the publication of the results (including negative results) in a relevant scientific journal.

The publication of the results should take into account the CONSORT statement and the FAIR Data principles. This means that the original data for the publications should be made available for subsequent use. The rights of third parties, in particular data protection and copyright, must be respected. These publications must be submitted to us as evidence. In all cases, the registration number of the study must be listed alongside the BMBF funding code.

If it can be proven that the manuscripts can not to be published in a specialist journal, they must be made publicly available in a WHO-certified primary register.

Reporting obligations

The annual interim reports to be submitted in accordance with No. 4.4 NABF should not exceed 4 pages. The report must be accompanied by a statement from the independent data monitoring committee (DSMB) stating that there are no concerns about the continuation of the study.

In addition to the annual report, quarterly reports must be submitted from the start of the term on January 31, April 30, July 31 and October 31 (and if necessary additionally on request) to report on the current status of patient recruitment using the attached table (see attached sample). If recruitment has not yet begun, a short report on the status of the work must be submitted to us by the dates mentioned. This report must provide information on the points listed under "Documents to be submitted".

Study audits

We reserve the right to carry out study audits.

Contracts with study centers

We would like to point out that contracts must be concluded with all participating study centers, which state the type and extent of the cooperation as well as details of the reimbursement of expenses.

Restitution of the grant

We reserve the right to temporarily reclaim partial grant amounts paid on request if they are not used by you on time.

Overpayment

Any losses that arise after the project has been completed must be repaid by you immediately and without being asked to do so, stating a reference number that will be communicated to you separately. Please note that a reference number can only be used once and must be requested from us before you transfer the money so that a payment can be allocated.

Please use the following bank details:

Recipient/account holder: **Bundeskasse Halle**

Deutsche Bundesbank, Leipzig branch

IBAN: DE38 XXXX XXXX XXXX XXXX 40

BIC: MARKDEF1860

The interest to be paid in accordance with Section 49a Paragraph 3 of the Administrative Procedure Act must be transferred to the aforementioned Federal Treasury account, stating a cash register number that will be communicated separately for this purpose.

Requirements for payment of the grant

The grant can only be paid out when the decision has become final after the expiry of the legal remedy period and all other requirements have been met. You can bring about the finality of the grant decision beforehand if you declare on the “Waiver of legal remedy” form that you waive an appeal (form is enclosed).

The form for requesting or calling up the grant in accordance with No. 2.5.1 NABF is already enclosed with the grant decision, provided that the payment plan provides for a payment in the current financial year. If you do not waive the right to lodge an appeal, you must wait until the appeal period has expired and enclose a declaration with the first payment request that you have not lodged an appeal.

We have sent a copy of the decision to the project management and the responsible third-party funding administration for their information.

3. Legal remedy instructions

An appeal can be lodged against this decision within one month of notification. The objection must be lodged with the **German Aerospace Center, DLR Project Management Agency, Heinrich-Konen-Straße 1, 53227 Bonn**.

With kind regards

i. A.	i. A.
Dr. Wrobel	Dr. Peters

This notice was created electronically and therefore does not bear a signature.
